# The Nature of Unconscious Attention to Subliminal Cues

**DOI:** 10.3390/vision3030038

**Published:** 2019-08-01

**Authors:** Seema Prasad, Ramesh Kumar Mishra

**Affiliations:** Center for Neural and Cognitive Sciences, Science Complex, University of Hyderabad, Hyderabad, Telangana 500046, India

**Keywords:** attention, unconscious, subliminal, top-down, bottom-up, contingent capture

## Abstract

Attentional selection in humans is mostly determined by what is important to them or by the saliency of the objects around them. How our visual and attentional system manage these various sources of attentional capture is one of the most intensely debated issues in cognitive psychology. Along with the traditional dichotomy of goal-driven and stimulus-driven theories, newer frameworks such as reward learning and selection history have been proposed as well to understand how a stimulus captures attention. However, surprisingly little is known about the different forms of attentional control by information that is not consciously accessible to us. In this article, we will review several studies that have examined attentional capture by subliminal cues. We will specifically focus on spatial cuing studies that have shown through response times and eye movements that subliminal cues can affect attentional selection. A majority of these studies have argued that attentional capture by subliminal cues is entirely automatic and stimulus-driven. We will evaluate their claims of automaticity and contrast them with a few other studies that have suggested that orienting to unconscious cues proceeds in a manner that is contingent with the top-down goals of the individual. Resolving this debate has consequences for understanding the depths and the limits of unconscious processing. It has implications for general theories of attentional selection as well. In this review, we aim to provide the current status of research in this domain and point out open questions and future directions.

## 1. Introduction

We are constantly bombarded by a variety of stimuli in our daily life. At any given time, only some of it is relevant; a lot of other information may not be useful to us in any way. For instance, when you are reading a book, you need to focus on the text and not get distracted by your flatmate speaking loudly on the phone. How successfully can we ignore irrelevant objects? The last 30 years or so has witnessed a heated debate on this topic with some studies showing that the stimulus properties solely determine where and how attention is allocated (your flatmate speaking on the phone will inevitably distract you) and others proposing that attentional selection is contingent on the top-down goals of the individual (you will not get distracted if your goal is to read the book). This “attention capture debate” has mostly been restricted to attentional selection of consciously perceived information (e.g., [1,2,3,4]). However, attention can also act on unconscious information [5]. Much like the debate with consciously perceived cues, the unconscious attention capture debate (although not as extensively studied) has also been riddled with opposing viewpoints with no solution in sight.

For example, in one of the first studies to comprehensively demonstrate top-down attentional control of unconscious cues, Ansorge and Neumann [6] conclude that “the results confirm the notion of contingent attentional capture and extend previous finding to show that contingent capture also holds for nonconsciously registered information (p. 774)”. In complete contrast, Schoeberl, Fuchs, Theeuwes, and Ansorge [7] claim that “the current findings unequivocally provide evidence that subliminal attentional capture is completely stimulus-driven (p. 746)”. There is no clarity on why and how such differing viewpoints have arisen on a single phenomenon. In this article, we will first review the key findings regarding the relative contributions of stimulus-driven and goal-directed factors in attentional selection of unconscious stimuli. We will then address the discrepancies among these findings and propose possible explanations that might help resolve the debate. Specifically, we examine the evidence for the answer to the following question: can we selectively attend to information that is relevant to us, even when we are not consciously aware of such information?

Before getting into the question of top-down attentional selection of unconscious information, it is first and foremost necessary to examine whether it is at all possible to attend to objects in the absence of conscious awareness. Answering this question requires us to examine the relationship between attention and awareness, which is one of the most widely debated topics in cognitive psychology. Throughout this article, we will use “consciousness" and “awareness” interchangeably to denote the accessibility or the report-ability of the contents of consciousness. Researchers have long been interested in whether the mechanisms involved in attention and awareness are completely independent of each other or whether there is any relationship between them [5,8,9,10,11,12]. Some have argued that attention and conscious awareness are inseparable and depend on each other [10]. That is, attention is necessary for conscious awareness and vice versa. Others have argued for a clear independence suggesting that it is possible to attend to objects without being conscious of them and to be conscious of objects without attending to them [5]. Attentional modulations of unconscious stimuli provide strong evidence for such a dissociation between attention and consciousness at least in one direction—that we can attend to objects in the absence of conscious awareness.

## 2. Attentional Control Over Unconscious Processing

It has long been assumed that the processing of unconscious information proceeds in an “automatic” manner, independent of the goals of the individual. Strategic processing and cognitive control is assumed to be exclusive to the stimuli that are consciously perceived. This alleged association between automaticity and lack of consciousness finds its origins in some of the classic theories of attention and control [13,14,15,16]. For instance, in a highly influential paper, Posner and Snyder [15] argued that a process can be termed automatic if it fulfils the following criteria: (1) it is initiated without any deliberate intention; (2) it cannot be interfered with by external processes; and (3) it proceeds in the absence of conscious awareness. Bargh [17,18] similarly showed that any process can be termed automatic if it includes the following “four horsemen of automaticity”—efficient, unintentional, uncontrollable and unconscious. Although there is no universal definition of automaticity (see [19] for a review) so far, lack of awareness has been a defining feature of automaticity according to most of the early theories. Thus, unconscious processing has been considered to be a proto-typical example of an automatic process and the influence of this view can be seen in many areas of cognitive psychology—even today.

It is only recently that this view has been challenged in favour of newer theories of automaticity which posit that automatic processing in general depends on the configuration of the cognitive system and is susceptible to currently-active task intentions. That is, even automatic processes critically depend on higher-level, top-down factors such as attention, task-intentions and goals. This has led to more refined ideas which allow for flexible top-down control over the processing of unconscious information (e.g., [20,21]). The processing of such unconscious information is flexible in the sense that a given attentional or goal-state is necessary for unconscious stimuli to trigger further processes. However, they are “automatic” in the sense that once initiated, they proceed without awareness and are no longer under any intentional control. Thus, the control settings have to be specified in advance in the case of unconscious information—often known as pro-active control. This is in contrast to top-down control on conscious information [22] which is susceptible to both pro-active (specified in advance) and reactive control (triggered dynamically in response to ongoing stimulus processing). Thus, it has been suggested that both unconscious and conscious information are susceptible to top-down control—but they differ in terms of the temporal onset of the control processes [23].

Unconscious processing can be studied in different ways. In this review, we will focus on the unconscious cuing of attention—where stimuli that are not consciously reportable influence subsequent orienting of attention. Such studies are also typically accompanied by an objective or subjective awareness test of the stimuli to verify that they were indeed unconscious. This dissociation between the processing of unconscious stimuli and its access to conscious awareness is the hallmark of establishing unconscious priming [24]. The existence of such unconscious influences on behaviour has been hotly debated in the past [25,26]. While the phenomenon is no longer outrightly rejected, the controversy has shifted to the depths and limits of unconscious processing. Specifically, researchers are now interested in examining which aspects of a stimulus can be processed unconsciously, what is the level of interaction possible in the absence of awareness and what control mechanisms can be initiated in the absence of conscious awareness.

The extent to which attention and other factors modulate unconscious processing has been examined primarily using two different paradigms: the masked priming paradigm [27,28] and variants of the spatial orienting paradigm [29]. The masked priming paradigm (also referred to as “subliminal priming paradigm” when the primes are shown to be objectively/subjectively unconscious) has been extensively used to probe the influence of an unconscious stimulus on response behaviour [30,31,32]. Typically, masked primes are presented for a very short duration followed by target stimuli. The targets (for example, the numbers 1 or 2) are arbitrarily assigned to responses (say A and L). Crucially, there can be a match or a mismatch between the responses triggered by the target and the prime. When the delay between the prime and target presentations is short, it is commonly found that responses are faster when both the prime and the target are associated with the same response: prime ‘1’ followed by target ‘1’. Such a paradigm—where a masked prime triggers a motor response—is typically known as the visuomotor priming paradigm. Semantic priming has also been demonstrated with subliminal primes where it is shown that the meaning of an invisible stimulus can also be accessed unconsciously. In a classic study, Marcel ([33] Experiment 4) presented a lexical decision task wherein two words were presented one after the other. The first word was pattern masked, energy masked or unmasked and the participants were not required to give any response. The second word was always unmasked and the participants had to judge whether it was a word or not. Marcel observed that participants were faster responding to the second word (BUTTER) if it was semantically associated with the first word (BREAD)—despite being unaware of the masked works. This demonstrated that semantic analysis of words can proceed without awareness. More recently, Kiefer and Spitzer [34] elucidated the time course of such an activation and observed that unconscious semantic activation decays rapidly after stimulus onset, in contrast to conscious semantic activation.

### 2.1. Spatial Orienting Studies with Unconscious Cues

Another line of research that has demonstrated the influence of subliminal cues on response behaviour are the spatial orienting studies using the Posner cuing paradigm [29]. In a typical experiment, on each trial a peripheral cue is flashed briefly on the screen followed by a target which requires a response (manual or ocular). The influence of the cue is usually measured in terms of the cuing effect which is the difference in response times (RTs) on invalid (when cue and target locations do not match) and valid (when cue and target locations match) trials. If the delay between the cue and target is short (usually less than 100 ms), the cue facilitates responses to the target appearing at the cued location, resulting in faster RTs on valid trials. At longer delays, the pattern is reversed and is referred to as inhibition-of-return (IOR; [35]).

McCormick [36] was the first study to use a Posner cuing task with exogenous cues below the threshold of conscious awareness. The aim of McCormick [36] was to examine the role of awareness in exogenous capture of attention. The argument was that if it can be shown that exogenous orienting can happen even without awareness, then it proves that it is indeed automatic. The theoretical antecedents to this line of thinking once again goes back to traditional theories of attention and control (e.g., [15]) which made a case for the automatic and unconscious nature of exogenous control of attention, contrasting it with deliberate, voluntary, endogenous attention. In his study, McCormick [36] presented cues either subliminally or supraliminally. Participants were told that the target would appear most often at the uncued location. Faster orienting to the target when the cue and target locations matched was seen for subliminal cues. For supraliminal cues, participants were faster when the target appeared at the expected location (that is, the uncued location) suggesting that participants were strategically able to reorient their attention to the expected target location based on the cue. In the absence of awareness, the attention capture at the cued location was more automatic.

Surprisingly, McCormick [36] failed to observe IOR at long stimulus-onset-asynchrony (SOA). Ivanoff and Klein [37] explained the absence of IOR by conducting a similar study, but with cue-report and no-report conditions. In the cue-report condition, participants were asked to report on whether they detected the presence of the unconscious cue on that trial. IOR was observed at long SOA (1005 ms) only in the no-report condition, but not in the cue-report condition. The inclusion of the cue-report task made the unconscious cues task-relevant and hence, part of the attentional set. Participants failed to disengage from the unconscious cues leading to the absence of IOR in the cue-report condition. This explained the lack of IOR in the McCormick [36] study as well because the participants in that study were additionally asked to report whether they had perceived the unconscious cue on each trial. Interestingly, Ivanoff and Klein [37] observed facilitation at short SOA (105 ms) only in the cue-report, but not in the no-report condition. The authors explained this finding by assuming that “early facilitation combined with early IOR leaving no net facilitation”. This did not happen in the cue-report condition because attention never disengaged from the cue location (hence the absence of IOR). These sets of results showed that unconscious guidance of attention is susceptible to attentional control settings.

Similarly, Mulckhuyse, Talsma, and Theeuwes [38] also showed that cues presented subliminally can exogenously capture attention. A single grey circle presented for 16 ms served as the subliminal cue. The target (a black dot within a grey circle) could be presented either left or right of a central grey circle. The subliminal cue was presented exactly at the target location or at the opposite location. The task was to press the SPACE bar as soon as the target was detected. A short (0 ms) or long (1000 ms) SOA between the cue and the target was used. At short SOA, the subliminal cues facilitated responses to target on valid trials (when cue location matched the target location) as opposed to invalid trials. However, the effect was reversed at long SOA (IOR). The authors argued that this showed exogenous capture by subliminal onset cues. It was considered exogenous for several reasons: the cue was uninformative, it did not resemble the target and gave no information regarding the appropriate response for the target. Further, inhibition of return is considered to occur only when attention has initially shifted reflexively to a location (and not voluntarily). Thus, the authors argued that finding IOR at long SOA can be considered as evidence for exogenous capture of attention at short SOA by the subliminal cues (however, see [39]). This was the first study of unconscious spatial cuing used to show the classic facilitation-followed-by-inhibition effect for unconscious cues. Subsequently, several studies claimed to have shown exogenous attention capture by subliminal cues (see [40] for a review).

In contrast, Ansorge, Kiss and Eimer [41] claimed to show that subliminal stimuli can trigger goal-directed attention capture. The method used in this study was said to be based on the seminal study by Folk, Remington and Johnson [42] using the contingent-capture paradigm. In Folk et al. [42] participants searched either for a red coloured target among white distractors (colour singleton: a unique element in a display with homogenous items) or for a white cross in one of the locations (abrupt-onset: a sudden flashing of a stimulus for a brief duration). Crucially, before the target display, a cue was presented in any of the four possible target locations. The cue could be a colour singleton (one location surrounded by red dots and the others by white dots) or an onset cue (one locations surrounded by white dots). It was found that colour singleton cues captured attention when participants had to look for a colour target. Similarly, the onset cues captured attention when participants looked for an abrupt onset target. Thus, the current task goals of the participant determined what was selected for further processing. With these findings as the basis, in an ERP study, Ansorge et al. [41] presented a cue-display consisting of four circles for 17 ms. One of the colours matched the colour of the target to be searched for. The cue-display was followed by a target display on half of the trials, where the participants had to decide whether the coloured target was a diamond or a square. On the other half of the trials, no coloured target was presented. Participants were faster responding on trials when the cue location matched the target location. The key evidence came from the N2pc ERP component, which is a signature of attentional allocation to an item. Crucially, this component was observed for the target-coloured cues even when there was no target to be searched for. Based on this, the authors argued that the task-instructions (“look for x coloured target”) lead to goal-oriented attentional capture by the target-coloured cues, irrespective of whether the task was to be subsequently performed on that trial or not. This was taken as evidence for the claim that the top-down attentional set a priori determines selection priority, even for unconscious stimuli. However, one major difference between this study and the contingent capture studies such as Folk et al. [42] needs to be noted. Ansorge et al. [41] only used target-matching colour cues. In contrast, Folk et al. [41] used both mismatching- and matching-colour cues and the the key argument for top-down control in their study comes from the fact that cue-validity effects were seen for colour-cue-colour-target—but not for abrupt-onset-cue-colour-target (and vice versa for abrupt-onset cues). Thus, the cue either matched/mismatched the task-set, which was not the case in Ansorge et al. [41]. To what extent this difference raises questions regarding the claim by Ansorge et al. [41] for top-down control will be discussed in later sections (Section 3.3).

### 2.2. Unconscious Effects on Oculomotor Selection

Can unconscious cues influence the oculomotor system? Recent evidences have demonstrated a dissociation between awareness and eye movements [43], where eye movement behaviour can be modulated by information that is not consciously accessible. One of the earliest demonstrations for this came from studies on blindsight patients with lesions in visual cortex who make saccadic eye movements to visual stimuli that they were not aware of (e.g., [44]). Superior colliculus (SC) has been implicated in meditating such visually guided behaviour in the absence of awareness through retinal projections that directly provide input to the SC bypassing the primary visual cortex [45]. Such a mechanism possibly exists because full perceptual analysis of any visual information takes time; whereas actions often need to be performed fast—sometimes even without the observer being fully perceptually aware. Eye movements provide a fast, orienting response and are continuously updated to reflect changes in the visual environment. Eye movements are also initiated at a faster time scale than manual response times, and are thus more susceptible to subtle modulations by invisible stimuli.

The influence of unconscious cues on eye movements was examined in a study by Van der Stigchel, Mulckhuyse, and Theeuwes [46]. Participants had to make a vertical saccade to a target (a diamond) placed either above or below the fixation in the presence of four placeholder circles on the screen. A subliminal cue was presented for 17 ms at one of the four locations just before the target screen. It was observed that subliminal cues in the opposite vertical field deviated the saccade trajectory to the target. This and similar other studies [47] were taken as evidence for the bottom-up capture of eye movements by subliminal cues. Weichselbaum, Fuchs, and Ansorge [48] argued that in [46] both the target and the cue were of the same contrast polarity (light grey cue/target against grey background) and thus the effects could be because of top-down capture. That is, since participants were expected to look for a light grey target, the light grey subliminal cues could have captured attention in a contingent manner because of this match. To test this hypothesis, Weichselbaum et al. [48] repeated the study with a specific manipulation: subliminal cues of two different contrast polarities were used. The contrast polarity of the cues either matched (e.g., white cues and white targets) or did not match (e.g., white cues and black targets) the contrast polarity of the targets. If subliminal cues capture attention in a top-down manner, then cues of mismatching contrast polarity should not affect eye movements. Instead, if subliminal capture is bottom up, then the contrast polarities should make no difference. The results showed that saccade latencies to the target were slowed down by a subliminal distractor in the opposite field. Crucially, this effect was observed for both contrast polarities which the authors claim as evidence for the bottom-up nature of subliminal capture.

## 3. Possible Explanations for the Inconsistencies

All the studies—to the best of our knowledge—that have used variants of the Posner cuing paradigm with unconscious spatial cues and their relevant details have been tabulated in Table 1. As is evident from the table and the review of literature presented so far, the evidence for goal-oriented processing of unconscious stimuli has been mixed [40,49] within the spatial orienting paradigm. In contrast, evidence for top-down control of unconscious processing is more consistently found with the masked priming paradigm where top-down attention is mostly defined on the basis of current task-sets [20,21,50]). It is important to note that these two paradigms are designed to measure different cognitive processes. The masked priming paradigm measures the extent of unconscious influences on responses where the masked primes contain information relevant to the required response. In contrast, the spatial cuing paradigm with unconscious cues measures the orienting of attention to exogenous subliminal cues. These cues may or may not be informative regarding the location or other relevant features of the target. Below, we only review the findings obtained from the spatial cuing paradigm, attempt to fill the gap between them and reconcile the contradictory results. It is to be noted that we are only focusing on studies with unconscious spatial (peripheral) cues and will not be reviewing studies with masked central cues.

### 3.1. Top-Down Control over Unconscious Stimuli Is More Prominently Seen with Feature-Based Attention, Than with Spatial Attention 

It is widely accepted that there are two major forms of attention: spatial and feature-based attention [51]. Spatial attention refers to the selection of particular locations in space whereas feature-based attention controls selection of particular attributes (colour/shape) of an object. These two forms of attention operate differently and are based on distinct neural mechanisms [52,53]. It is not clear which of these forms of attention is more susceptible to top-down control by goal-relevant information. Spatial attention is also known to be closely linked with the saccadic system. We constantly move our eyes—either based on where we want to look or because something in the visual field caught our eye due to its salience. This sensitivity to salient stimuli in the environment has been evolutionarily important to us in order to avoid sudden dangers in our environment. Studies that have examined the time-course of spatial vs. feature-based attention have found that the effects of feature-based attention is often delayed, whereas spatial-attention acts during the early stages of visual processing [54]. It is well known in the attention literature that slow, delayed responses reflect volitional, goal-driven processes [55] because it takes time to implement higher-order control. Similarly, based on single-neuron recordings, Hayden and Gallant [53] concluded that spatial attention is driven by both bottom-up and top-down factors whereas feature-based attention is mostly goal-driven (although this is not applicable to singleton or contrast detection which is mostly stimulus-driven, [56,57]). Thus, it might be argued that unconscious processing is perhaps more susceptible to top-down modulation when attentional deployment is feature-based. In support of this idea, Kanai, Tsuchiya, and Verstraten [58] found that top-down feature-based attention modulated the perception of invisible stimuli whereas spatial attention did not. This could be one of the reasons why masked priming studies that manipulate feature-based attention to the masked primes have predominantly found evidence for top-down control of unconscious priming, whereas the findings from spatial cuing paradigm is less consistent.

Few studies have been able to bridge this gap between masked priming and spatial cuing [6,59,60]. For instance, Scharlau and Ansorge [60] administered the temporal-order judgement task (ToJ) in which the participants were presented two stimuli (diamond and a square) with a short asynchrony and were asked to report which of the stimuli appeared first. It was found that a masked prime (a smaller replica of the target) facilitated the conscious perception of the stimulus at the primed location. This effect, known as perceptual latency priming (PLP), is considered to be attentional in nature and is said to arise from shifts of attention to the primed location ([61,62,63], however, see [64] for alternative non-attentional explanations of ToJ task). Crucially, Scharlau and Ansorge [61] observed that PLP was greater when the prime features matched the target features than when they matched the non-target features, suggesting that attention is captured more by unconscious information that match the participant’s current task-goals. Thus, top-down attentional effects of unconscious stimuli have been seen not just on response times, but also on the latency of conscious perception.

In another classic study, Ansorge and Neumann [6] used masked primes within the peripheral cuing paradigm and comprehensively demonstrated the role of task-intentions on unconscious priming effects. The target display in this study was an array of two rectangular boxes arranged horizontally. Horizontal bars above and below one of the boxes marked the target. The prime display was a replica of the target display, but with smaller boxes. Similar to the target display, horizontal bars above/below one of the boxes marked the prime. The task was to search for a coloured target and respond based on its location. The unconscious primes were presented either left or right of the fixation cross. The targets could be presented at the primed location or in the opposite location. Priming effects were observed when the prime matched the current task-intentions. That is, a red-coloured prime (but not a black-coloured prime) resulted in priming effects when the task was to look for a red target. Thus, the task-goals are said to selectively enhance certain features of the masked primes depending on their relevancy to the task. This is in line with the findings from contingent-capture studies which demonstrate that deployment of spatial attention depends on feature-based attentional control settings [42]. As can be seen from Table 1, all the studies that have demonstrated goal-oriented processing of unconscious primes (in bold) have used a variant of the feature-based cuing paradigm (except [37], where the unconscious cues matched the top-down goals in the cue-report condition). In these studies, a particular target feature is cued (mostly, colour) and the participants have to perform a discrimination task on the target [41,59,65,66,67]. Thus, it is possible that top-down attentional control on unconscious priming is seen mostly when attentional deployment is feature-based—even within a spatial orienting paradigm.

Some researchers argue that the effects seen in feature-based attentional paradigms such as the contingent-capture paradigm can be attributed to inter-trial priming and not necessarily to top-down control ([68,69], however, see [70]). In Folk et al. [42], for instance, participants searched for a specific type of target for an entire block, which could have led to bottom-up inter-trial priming. That is, the target of the previous trial could have primed the target of the current trial. To test whether contingent capture is seen even when the trials (and target-types) are mixed, Belopolsky, Schreij and Theeuwes [69] replicated the Folk et al. study with a minor change—participants were cued before each trial to look for either an abrupt-onset target or a colour-target. Thus, the type of target could change from trial-to-trial and was not constant across a block of trials. The results showed no evidence for contingent-capture. This and other similar findings [71,72,73] have lead researchers to argue that so-called top-down effects seen in such feature-based attentional studies could originate from the establishment of attentional set as a result of previous (trial) experiences with the target and might have nothing to do with top-down control. A similar argument can be made for unconscious cuing studies showing contingent-capture [6,41]. Thus, it is possible that task-goals played little to no role in these studies and the observed effect could be attributed to repeated exposure to the same type of target leading to bottom-up activations associated with the target. To provide definitive evidence for establishment of top-down attentional sets, it is necessary to have mixed designs where the task-relevancy or task-goal changes dynamically from trial to trial. Such a design rules out contributions from inter-trial priming and can provide strong support for strategic, top-down attentional control over unconscious attention.

### 3.2. Abrupt-Onset Cues Automatically Capture Attention

Abrupt onset cues have been extensively used to study unconscious cuing. Most such studies have observed attention capture by cues irrespective of task-goals (e.g., [38,46] lending support to the claim that unconscious attention is primarily stimulus-driven. In fact, many have argued that supraliminal abrupt-onset cues are a special category of stimulus that capture attention automatically irrespective of task-goals [74]. In contrast, goal-driven theories have argued that abrupt-onset cues capture attention when the task is to respond to an abrupt-onset target [42,75]. In these cases, the abrupt-onset cues become part of the participants’ attentional set thereby facilitating capture. Recently, Gaspelin, Ruthruff, and Lien [76] provided a parsimonious explanation of these various contradictory findings regarding the nature of attention capture by irrelevant supraliminal cues. They proposed that abrupt onset cues always capture attention, but the extent to which attention dwells on the cues and manifests as cuing effects depends on the difficulty of the task. When the search for the target is easy (e.g., to look for a red target among blue or green distractors), the abrupt onsets that do not match the top-down goals are rejected more easily and cuing effects are lesser. However, when the search is difficult (search for E among other letters), it is harder to decide whether the cue is really a distractor and thus the cue is slowly rejected resulting in increased cue effects. In short, the authors argued that evidence for bottom-up capture by abrupt onset cues is more commonly found in studies with a difficult search, because an easy search is an insensitive measure of attention capture.

It is to be noted that the attentional dwelling hypothesis is formulated based on the modified spatial cuing paradigm, where the task is to search for a target in the presence of distractors. Most studies with abrupt-onset unconscious cues have used detection or localisation tasks ([38,77,78,79,80] Experiments 1–3). The predictions of the attentional dwelling hypothesis can be tested on unconscious cues in a study where the task is to search for a target—which is either easy or difficult. For instance, in Fuchs and Ansorge ([77] Experiments 4 and 5), participants had to look for a colour-defined target (red among green and blue) following a subliminal abrupt-onset cue (in black colour). The authors observed no cuing effects indicating that the cues completely failed to capture attention because of the mismatch between the cue and target colours. This was taken as evidence that abrupt-onset cues do not necessarily always capture attention. However, based on the attentional dwelling hypothesis one could argue that because the study used an easy colour search, attentional dwelling effects at the cued location were masked resulting in much smaller, if not no cuing effects. The same study with a difficult letter search might have given rise to cuing effects, suggesting that attention-capture by the subliminal cues in this study was stimulus-driven and not goal-driven. Ansorge and Neumann [6] similarly used an easy colour search with abrupt-onset cues to argue for goal-driven modulation of the processing of unconscious cues.

Future studies are required to definitively assess whether the implications of the attentional dwelling hypothesis is also valid for unconscious attention capture. It is necessary to conduct studies in which the search difficulty is manipulated in a modified spatial cuing paradigm with abrupt-onset cues. Several studies have shown that colour cues are relatively less efficient in capturing attention in a bottom-up manner compared to abrupt-onset cues [81]. In line with this, most studies that have found evidence for goal-driven attention capture with unconscious cues have used colour singleton cues in a colour search task ([6,41,59,65,66,67] Experiment 5). It is possible that search difficulty plays a role in attention capture by subliminal onset cues, but not for colour cues. A single study comparing both in a paradigm designed to test the dwelling hypothesis will throw more light on how the type of subliminal cue plays a role in the orienting of unconscious attention.

### 3.3. Inclusion of Both Matching and Non-Matching Cues

What establishes the involvement of top-down control in attention capture? Conversely, when is attentional capture considered to be truly exogenous? For instance, attentional orienting in Posner cuing paradigm with abrupt peripheral cues has been considered the hallmark of exogenous, stimulus-driven attention capture. This is primarily because the abrupt cues are spatially uninformative of the target location. However, even though a sudden onset cue is spatially uninformative, it still matches the top-down goals of the participant when the task is to look for an onset singleton. For instance, the participants in Van der Stigchel et al. [46] were asked to make a saccade to a singleton target. The subliminal cue was a grey circle, which was also a singleton. The participants can be said to adopt a singleton detection mode [82] where the presence of a singleton cue captures attention as it is part of the task-set to look for a singleton target. This criticism is applicable to many of the studies tabulated in Table 1 where both the cue and target were abrupt-onsets. Thus, the findings of Van der Stigchel et al. [46] and several subsequent studies ([48,59,78] Experiments 1–3), do not reflect purely stimulus-driven capture as they can be explained through top-down contingent attentional capture.

Schoeberl et al. [7] acknowledged this limitation of previous findings and addressed it in a study where they presented cues that were truly non-matching with the top-down goals and examined whether they facilitated the search for a colour target. The authors ensured that cues did not have any matching feature with the target by pairing a colour target with a singleton cue. If the contingent capture account is valid for subliminal cues, the authors argued that non-matching cues should not lead to cuing effects. If the bottom-up account is valid, then cuing effects should be observed even though the cues did not match the goals of the participant. The authors found robust cuing effects which led them to conclude that attention capture by subliminal cues is “completely stimulus-driven”. The key difference between these studies and studies such as Ansorge et al. [41,65] is that Ansorge et al. [41,65] argued for the top-down contingent capture based on attention capture by matching cues, whereas the Schoeberl et al. [7] presented only non-matching cues. Is it possible that contingent capture is observed only when a matching cue condition is compared with non-matching cue condition? As can be seen from Table 1, all the studies so far that have found evidence for top-down contingent capture have compared matching and non-matching cues, except [41]—which only had a matching cue condition. However, even in the Ansorge et al. [41] study, the claim for top-down control may not be warranted because they only used target-matching colour cues. Not finding N2pc on mismatch trials would have been a strong evidence for contingent-capture. Since they did not have such a condition, it is not possible to draw firm conclusions on how effectively top-down control was exercised.

It is important to compare the effects of both types of cues to firmly establish their relative contributions to attention capture. For instance, Lamy et al. [66] conducted a study where a single subliminal colour cue either matched the target colour or the distractor colour. The task was to search for a target defined by its colour and report its orientation. They found that only target-coloured cues elicited cuing effects, indicating that attention capture is contingent on the participant’s attention set. Including both matching and non-matching cues is important because attention is primarily a selective process. Selection occurs in the face of competition—when different stimuli compete for capacity-limited resources of the brain. The attentional system is forced to select only relevant or important information when there is competition from irrelevant information. For instance, attentional effects are found to emerge when targets are presented along with distractors—but not when they are presented alone [83,84,85]. Thus, it is possible that goal-relevant unconscious stimuli are more likely to be preferentially processed when there is competition for selection from goal-irrelevant stimuli. In the absence of this competition, there is no need for the participants to engage their control mechanisms. Barring few exceptions (see also [65,86], not many studies have included both matching and non-matching cues. Further, in all these studies, match/mismatch has been manipulated at the feature-level (colour/shape, etc.). It has been known for long that spatial attentional control settings can influence attention capture by supraliminal cues ([87] Experiment 2). No study so far—to the best of our knowledge—has examined this in the context of unconscious attention. It is necessary to conduct more such studies involving feature-based or spatial match/mismatch between the cue and the target to understand the role of attentional control settings on unconscious cuing.

### 3.4. Attention Capture Can Be Triggered by Both Stimulus and Goal-Driven Factors—However, Attentional Engagement Is Goal-Driven

Attentional selection first involves the shift of attention to an object/location (“attention capture”) which is followed by enhanced processing of the information related to that object or location (“attentional engagement” [88,89]). More recent studies have shown that stimulus-driven and goals-driven factors might contribute differently to these two mechanisms. For instance, Zivony and Lamy [90] presented supraliminal abrupt-onset cues followed by a display of four letters. The task was to find the red-lettered target (e.g., “E”) among distractors (“E” and “H” in pink/orange) and press a button accordingly. The target could appear either at the cued location or the uncued location, giving rise to valid and invalid trials. Crucially, distractor compatibility effects were calculated when the cue and the target appeared in different locations. The trials for which the cued distractor shared the identity of the target letter (e.g., E, but in pink or orange) were termed as compatible trials. The trials for which the cued distractor was different than the target letter (e.g., H in pink/orange) were termed incompatible trials. Thus, the compatibility effects were an index of the extent to which the identity of the distractor was processed at the cued location—reflecting the degree of attentional engagement triggered by the cue. The authors found attention capture (cuing effect) by both target-matching and non-matching cues; whereas attentional engagement (distractor compatibility effect) was observed only with matching cues. Thus, the authors argued that whereas the initial shift of attention to a location can proceed in a bottom-up manner, further engagement with the objects at that location occurs only if those objects or their features are relevant to the current goals.

It should be noted that this study was conducted with supraliminal cues. Most of the studies using the spatial cuing paradigm with unconscious cues have measured cuing effects, and thus only the extent of attention capture but not attentional engagement is known. The crucial distinction made by Zivony and Lamy [90] is that shifts of spatial attention during capture are “shallow” and can occur irrespective of top-down goals. However, further enhanced processing of the identity of the object at the cued location is costly and occurs only if it is necessary for the task at hand. If top-down control is possible only for attentional engagement, this could explain the bottom-up unconscious capture observed in studies like Schoeberl et al. [7]. The unconscious cues in Schoeberl et al. [7] could have captured attention because the study only measured the extent of the shift in spatial attention to the cue. It is possible that non-matching cues would not have had any influence if the participants were required to process/reject the identity of the cue. There have been other such studies that presented both matching and non-matching abrupt-onset cues [48,78] and found similar cuing effects for both types of cues. However, again, these studies only measured the extent of attention capture and not engagement. Thus, it might be necessary to revise the existing ideas of top-down attentional control by making clear distinctions between attention capture and engagement.

This hypothesis may seem to be in contrast with the findings of Ansorge et al. [65] who compared the effects of target-matching and non-matching cues on a task that required the participants to search for a colour-defined target and discriminate based on the shape. The subliminal cues could either be congruent (with response-relevant shape information) or incongruent (response-irrelevant shape information) with the target and could be presented at the target location (valid trials) or in a different location (invalid trials). The authors observed cuing effects only for the target-matching colour cues and not for the non-matching cues, suggesting that non-matching cues failed to capture attention. It would have been reasonable to expect cuing effects for both matching and non-matching cues based on the earlier argument regarding attention capture. Instead, no evidence of capture by non-matching cues were observed. However, this only suggests that both stimulus-driven and goal-driven factors are capable of influencing attention capture. The key argument based on the Zivony and Lamy [90] study is that attentional engagement is solely goal-driven. Interestingly, no response congruency effects were observed at all in Ansorge et al. [65] thus failing to observe any evidence for engagement. Further, the response congruency in Ansorge et al. [65] was manipulated on both valid and invalid trials because their aim was to see if response congruency effects can be seen irrespective of shifts of attention triggered by the cue. In contrast, the compatibility effects in Zivony and Lamy [90] were calculated only on invalid trials. A replication study of Zivony and Lamy [90] with unconscious cues will shed further light on whether the above-mentioned differences between attention capture and engagement are also applicable for unconscious guidance of attention.

## 4. Neural Mechanisms of Top-Down Attention to Unconscious Stimuli

Is there any neural evidence for top-down selection of unconscious stimuli? As mentioned before, Ansorge et al. [41] found a significant N2pc (which reflects spatial shifts in attention, [91]) for the relevant subliminal cues. Using the continuous flash suppression paradigm, Travis et al. [67] recently replicated the contingent capture findings of Ansorge et al. [41] to find the neural correlates of top-down suppression of unconscious cues. Using a similar feature-based cuing paradigm, the authors found that only subliminal cues that matched the target colour were successful in orienting the participants’ attention to the cued location, thereby eliciting cuing effects. More importantly, authors found a significant N2pc for the target-colour cues and Pd (distractor positivity—a signature of distractor suppression, [92]) for the distractor coloured cues. The authors interpret the findings based on the signal suppression hypothesis [92,93] according to which top-down suppression mechanisms inhibit attention capture by distractor stimulus and enhance the processing of task-relevant stimuli. This position is a radical departure from earlier theories of visual selection, according to which attention is initially captured by salient distractors, and then redirected to task-relevant stimuli. Support for the signal-suppression hypothesis also comes from a recent study on monkeys, which used a combination of single neuron recordings and surface ERP measures [94]. Cosman et al. [94] found that neurons in the pre-frontal cortex and frontal eye field are responsible for both selection of relevant features and the suppression of irrelevant distractor items. Thus, the findings of Ansorge et al. [41] and Travis et al. [67] provides neural evidence of goal-oriented processing of relevant stimuli even in the absence of awareness.

Goal-directed processing of relevant stimuli or cognitive control has typically been associated with activations in the higher-order brain areas such as the pre-frontal cortex. Whereas such complex control of behaviour by the brain has been mostly restricted to the domain of consciousness [95], more recently, evidence has emerged that areas in the pre-frontal cortex can also be activated in response to unconscious stimuli [96,97]. Evidence has come from studies that have tracked the neural correlates of unconscious inhibition using tasks such as go no-go or stop-signal tasks, which tap into inhibitory control or conflict resolution [97,98,99,100]. Additionally, Ulrich, Adams, and Kiefer [101] showed dynamic functional connectivity between brain areas depending on the configuration of the task-set using fMRI. In a semantic categorisation task with subliminal primes, higher activation was found in regions responsible for semantic processing. This in turn led to enhanced processing of the semantic properties of the unconscious stimuli. This study provides neurobiological evidence for the attentional sensitisation model of unconscious cognition [21] and explains how the selective processing of unconscious stimuli based on their relevance to the current task goals is realised in the brain.

## 5. Conclusions

We have examined evidence for top-down attentional control of unconscious cuing based on studies from the spatial cuing literature. Our intention with this review was to bring out the inconsistencies in the unconscious cuing literature and offer possible explanations for them. It is not possible to offer a parsimonious explanation accounting for all the findings due to the paucity of the studies on this topic. We have examined which form of attention is more susceptible to top-down control (Section 3.1), if the studies claiming to have shown goal-driven (Section 3.2) or stimulus-driven (Section 3.3) attention capture have indeed done so, and the importance of distinguishing between attention capture and engagement (Section 3.4). Analysing the findings from all the studies covered in Table 1 suggests that evidence for top-down cuing effects with unconscious cues is more likely to be found when both matching and non-matching cues are presented on a task that typically involves some form of feature-search. However, most of such evidence comes from studies with colour singleton cues. All the studies with abrupt onset cues and abrupt-onset targets have found evidence for attention-capture which can be explained based on the contingent-capture account.

Our review has revealed that demonstration of top-down control over unconscious cuing depends on several crucial factors such as: the type of cue (abrupt onset vs. colour singletons), the relationship between the properties of the cue and the target features (whether they match or do not match) and finally, whether the task calls for attention capture or engagement. Teasing apart the individual contributions of each of these factors requires conducting experiments manipulating each of these factors with the same set of stimuli. Thus, it is necessary to conduct studies using a feature-based cuing paradigm involving both abrupt onset and colour singleton cues that match/ do not match the target features being searched for. Examining whether the differences in attention capture and attentional engagement can explain some of the inconsistent findings in the literature will be useful as well. While there are a handful of studies currently that have included one or more of these manipulations, it is necessary to conduct and replicate more studies to arrive at a comprehensive understanding. Additionally, especially in studies involving abrupt-onset cues, it is necessary to manipulate the search difficultly of the task and test the predictions of attentional dwelling hypothesis [76]. This will inform us whether unconscious abrupt-onset cues are indeed a special category of stimulus that capture attention automatically or if their effects can be explained based on the contingent capture account. There have also been other recent advances regarding the fate of irrelevant, distractor stimuli during visual processing. The signal suppression hypothesis [92], for instance, proposes that top-down signals (triggered by the setting up of the attentional set) proactively suppress the representation of irrelevant distractors, much before they capture attention. It is necessary to examine whether the signal-suppression hypothesis can also be extended to attentional selection of unconscious stimuli.

The evidence for top-down control over unconscious processing has far-reaching implications. Understanding the role of attention in the processing of unconscious stimuli assumes importance due to its potential in throwing more light on the relationship between attention and awareness. It is one of the most crucial pieces of evidence for the dissociation between attention and awareness as it illustrates that it is possible to attend to objects in the absence of conscious awareness. Demonstrations of top-down unconscious attention can also inform us regarding the nature of cognitive control and its dependence (or the lack of it) on conscious awareness. Importantly, it shows that unconscious processing is flexible and can adapt to the requirements of the individual.

## Figures and Tables

**Table 1:** Studies examining the nature of attentional control over unconscious processing using variations of the spatial orienting paradigm.

Sl No.	Study	Cue	Target	Task
[36]	McCormick (1997)	Abrupt onset	Abrupt onset	Discrimination
[79]	Leuthold and Kopp (1998)	Abrupt onset	Abrupt onset	Localisation
[37]	Ivanoff and Klein (2003)	Abrupt onset	Abrupt onset	Go/No-go
[38]	Mulckhuyse, Talsma, and Theeuwes (2007)	Abrupt onset	Abrupt onset	Detection
[6]	Ansorge and Neumann (2005)	Abrupt onset (Colour: match/mismatch)	Colour-defined target	Search followed by localisation
[41]	Ansorge, Kiss and Eimer (2009)	Colour singleton (Colour: match)	Colour-defined target ^1^	Search followed by discrimination
[65]	Ansorge, Horstmann, and Worschech (2010)	Colour singleton (Colour: match/mismatch)	Colour-defined target	Go/No-go followed by discrimination
[46]	Van der Stigchel, Mulckhuyse, and Theeuwes (2009)	Abrupt onset	Abrupt onset	Localisation (eye movement)
[59]	Held, Ansorge, and Mueller, 2010 (Experiments 1–4)	Feature-singleton	Feature-singleton	Localisation
[59]	Held, Ansorge, and Mueller, 2010 (Experiment 5)	Colour singleton (Colour: match/mismatch)	Colour- and shape defined target	Search followed by discrimination
[47]	Mulckhuyse and Theeuwes, 2010	Abrupt onset	Abrupt onset	Search followed by localisation (eye movement)
[39]	Fuchs and Ansorge, 2012a (Experiments 1–4)	Abrupt onset (contrast polarity: match/mismatch)	Abrupt onset	Detection
[77]	Fuchs and Ansorge, 2012b (Experiments 1–3)	Abrupt onset (contrast polarity: match/mismatch)	Abrupt onset	Detection
[77]	Fuchs and Ansorge, 2012b (Experiments 4 and 5) ^2^	Abrupt onset (Colour: mismatch)	Colour-defined target (non-singleton in Expt 4 and singleton in Expt 5)	Search
[77]	Fuchs and Ansorge, 2012b (Experiments 6) ^2^	Colour singleton (Colour: mismatch)	Colour-defined target	Search
[78]	Fuchs, Theeuwes, and Ansorge, 2013(Experiments 1 and 2)	Abrupt onset (contrast polarity: match/mismatch)	Abrupt onset	Detection
[78]	Fuchs, Theeuwes, and Ansorge, 2013(Experiment 3)	Abrupt onset (contrast polarity: match/mismatch)	Abrupt onset	Go/No-go
[48]	Weichselbaum, Fuchs, and Ansorge, 2014	Abrupt onset (contrast polarity: match/mismatch)	Abrupt onset	Detection (eye movement)
[7]	Schoeberl, Fuchs, Theeuwes, and Ansorge (2015)	Abrupt onset	Colour-defined target	Search followed by discrimination
[66]	Lamy, Alon, Carmel, and Shalev (2015)	Colour singleton (colour: match/mismatch)	Colour-defined target	Search followed by discrimination
[80]	Schoeberl and Ansorge (2018)	Abrupt onset (temporally predictive/unpredictive)	Colour-defined target	Search followed by discrimination
[67]	Travis, Dux, and Mattingley (2018)	Colour singleton (colour: match/mismatch)	Colour-defined target	Search followed by discrimination
[86]	Schoeberl, Ditye and Ansorge (2018)	High or low frequency cue (frequency: match/mismatch)	Frequency-defined target	Search followed by discrimination

Note: the studies that found (or claimed to have found) evidence for top-down attentional capture are in bold. The text inside the brackets in the “Cue” column mention whether both matching and non-matching cues or only one of them was presented. The dimension along which the properties of the cue (colour or contrast polarity etc.) matched or mismatched the target properties is also mentioned. ^1^ Unless specified, “colour-defined target” refers to a non-singleton target. ^2^ No cuing effects observed.
